# Swept-Source Optical Coherence Tomography in the Diagnosis and Monitoring of Optic Nerve Neuropathy in Patients with Wernicke’s Encephalopathy Due to Hyperemesis Gravidarum

**DOI:** 10.3390/jcm14113849

**Published:** 2025-05-30

**Authors:** Magdalena Kal, Michał Brzdęk, Justyna Tracz, Paweł Szadkowski, Dorota Zarębska-Michaluk

**Affiliations:** 1 Collegium Medicum, Jan Kochanowski University, 25-317 Kielce, Poland; kalmagda@gmail.com; 2 Ophthalmic Clinic of the Voivodeship Hospital, 25-736 Kielce, Poland; 3 Neurology Clinic, Swietokrzyskie Neurology Centre, 25-317 Kielce, Poland; justyna_t@o2.pl (J.T.); pawszadkowski@gmail.com (P.S.); 4 Department of Infectious Diseases and Allergology, Jan Kochanowski University, 25-317 Kielce, Poland; dorota1010@tlen.pl

**Keywords:** optical coherence tomography, optic nerve neuropathy, Wernicke’s encephalopathy, hyperemesis gravidarum

## Abstract

**Objectives**: This review explores the role of swept-source optical coherence tomography (OCT) in diagnosing and monitoring optic nerve neuropathy in Wernicke’s encephalopathy (WE) due to hyperemesis gravidarum, including a case of neuropathy from intractable vomiting in pregnancy. **Methods**: A literature search was conducted in the PubMed database to select high-quality reviews and original articles on the use of swept-source OCT for assessing optic nerve involvement in WE due to hyperemesis gravidarum. **Results**: WE is a potentially fatal neuropsychiatric syndrome caused by thiamine deficiency due to various causes, like alcoholism, malnutrition, and prolonged parenteral nutrition. This condition can cause neurological disorders such as imbalance, altered mental status, nystagmus, and ophthalmoplegia. Sometimes, there is also a deterioration of visual acuity with swelling of the optic disc. OCT is a non-invasive imaging tool that can detect optic nerve involvement in WE by assessing peripapillary retinal nerve fiber layer (pRNFL) thickness. In the acute phase, optic disc edema and increased pRNFL thickness may be observed, while chronic-phase changes include optic nerve atrophy and pRNFL thinning. WE may occur in the course of hyperemesis gravidarum in pregnant women. We present a case of a 23-year-old woman at 14 weeks of gestation with WE due to severe hyperemesis gravidarum, manifesting as visual impairment and neurological deficits. MRI confirmed the diagnosis, while OCT revealed transient pRNFL thickening followed by optic nerve atrophy. **Conclusions**: Early diagnosis and thiamine supplementation are crucial to preventing severe complications. OCT is a valuable tool for detecting and tracking optic nerve changes in WE.

## 1. Introduction

Wernicke’s encephalopathy (WE) is an acute neurological disorder caused by thiamine deficiency, impacting both the peripheral and central nervous systems. It can result from various factors, including alcoholism, severe malnutrition, gestational vomiting, prolonged parenteral nutrition, and conditions like chronic liver disease, hyperthyroidism, and anorexia nervosa. Infections and excessive carbohydrate intake in the presence of thiamine deficiency can also trigger WE [1,2,3].

The prevalence of WE remains difficult to determine due to challenges in diagnosis and underreporting. Autopsy studies suggest a prevalence ranging from 1% to 3%, while clinical records often indicate lower rates, reflecting the frequent misdiagnosis of the condition [4,5]. WE is more commonly observed in populations with nutritional deficiencies, particularly in regions where malnutrition is widespread [5]. The condition appears to be more prevalent in males than females, with an estimated ratio of 1:1.7 [6].

The clinical presentation of WE is typically characterized by a triad of ophthalmoplegia, ataxia, and mental status changes. However, not all patients exhibit all three symptoms, complicating diagnosis. Other neurological manifestations may include nystagmus, confusion, and peripheral neuropathy. If untreated, WE can progress to coma or death [7].

WE should be differentiated from Korsakoff syndrome, which may result from one or more episodes of WE. Korsakoff syndrome is characterized by significant anterograde and retrograde memory deficits, while immediate memory remains intact. Patients show impaired short-term memory with preserved consciousness and sensorium. Confabulation is a key symptom, occurring either spontaneously or in response to provocation. Spontaneous confabulations are more common in acute WE, while provoked confabulations are typical of Korsakoff syndrome [1,8].

The aim of this review article is to explore the role of swept-source optical coherence tomography (SS-OCT) in the diagnosis and monitoring of optic nerve neuropathy in patients with WE due to hyperemesis gravidarum. In addition to reviewing the current state of knowledge, we present a case of optic nerve neuropathy in patients with WE caused by intractable vomiting during pregnancy.

## 2. Materials and Methods

A Boolean search of the PubMed database was conducted to identify original studies and case reports published between January 2020 and November 2023 on the use of swept-source OCT in diagnosing and monitoring optic nerve involvement in WE due to hyperemesis gravidarum. The keywords used included “swept-source optical coherence tomography”, “optic nerve neuropathy”, “Wernicke’s encephalopathy”, “thiamine deficiency”, “ocular involvement in WE”, and “optic nerve head”.

Selection criteria encompassed relevance, methodological quality, relation to the research topic, number of citations, reported cases, and innovative methodologies. The most relevant articles were chosen for review, with a focus on key aspects, such as the role of thiamine (vitamin B1), the mechanism of optic nerve neuropathy in WE, ocular involvement in WE, diagnosis and treatment of WE, prognosis, and the utility of OCT in detecting optic neuropathy.

## 3. Role of Thiamine (Vitamin B1)

Thiamine is a water-soluble vitamin found naturally in animal and plant products. The body’s requirement for this vitamin is directly related to total calorie intake [9]. The average daily requirement for thiamine is approximately 1.5 mg, and its demand increases in hypermetabolic states such as pregnancy [10].

Thiamine plays a crucial role in carbohydrate metabolism, particularly in the decarboxylation of alpha-keto acids. It serves as a coenzyme for the enzyme transketolase in the glucose pentose monophosphate pathway, which is essential for energy production and nervous system function. Deficiency of thiamine can result from inadequate intake, impaired absorption, increased metabolic demands, or inefficient utilization [11]. Thiamine requirements increase during pregnancy, probably as a result of sequestration of the vitamin by the fetus and placenta [12]. In cases of hyperemesis gravidarum, persistent vomiting exacerbates thiamine deficiency by further impairing nutrient absorption. If left untreated, this can lead to severe neurological complications, including WE [13].

Thiamine levels should be measured in whole blood rather than plasma, as most circulating thiamine is protein-bound. Free thiamine concentrations in plasma and urine do not accurately reflect tissue stores, making them unreliable for diagnostic purposes [14]. Laboratory assessment of thiamine deficiency often involves high-performance liquid chromatography, which provides precise measurements but may take several days to yield results [15]. Given its vital role in neurological and metabolic functions, the early recognition and correction of thiamine deficiency are essential to prevent irreversible damage, particularly in vulnerable populations such as pregnant women experiencing severe vomiting.

## 4. Diagnosis of WE

The diagnosis of WE is primarily clinical, as there are no definitive laboratory tests to confirm the condition [16]. The classic triad of symptoms includes disorientation (63%), ocular abnormalities (57.1%), and ataxia (82%), though these features are present in only 47% of cases [17]. Given the variability in clinical presentation, a high index of suspicion is necessary for timely diagnosis and treatment.

The diagnostic criteria for WE require at least two of the following features [7]:

- Ocular abnormalities (e.g., ophthalmoplegia, nystagmus, ptosis).
- Dietary deficiency of thiamine.
- Altered mental status (confusion, memory impairment, or coma).
- Cerebellar dysfunction (ataxia, unsteady gait).

Blood tests to measure thiamine levels can support the diagnosis, but no universally accepted critical threshold below which WE occurs exists. Not all patients with WE exhibit low thiamine levels, and their absence does not rule out the condition. A complete blood count and metabolic panel should be conducted to exclude other causes of neurological impairment [16].

Brain MRI is the most valuable test in making a diagnosis of WE due to its high specificity [18]. Brain MRI detects WE-typical lesions in half to two-thirds of cases. Typical MRI findings include hyperintense signals on FLAIR and T2-weighted images in the periventricular thalamus, mammillary bodies, and periaqueductal gray matter [15,19]. However, normal imaging does not exclude WE, as the condition remains a clinical diagnosis [20].

Additional biochemical assessments, such as erythrocyte transketolase activity and lactate/pyruvate levels, can indicate thiamine deficiency. Thiamine serves as a cofactor for pyruvate dehydrogenase, and its deficiency leads to elevated lactate and pyruvate concentrations. However, these tests are not routinely performed due to their limited availability [21].

Since WE is a medical emergency, prompt recognition and treatment with thiamine supplementation should not be delayed while awaiting confirmatory test results. Early intervention is crucial to prevent irreversible neurological damage and progression to Korsakoff syndrome [7].

## 5. Treatment and Prognosis of WE

The primary treatment for WE is thiamine replacement, which should be administered promptly to prevent irreversible neurological damage. Traditionally, a parenteral dose of 100 mg of thiamine per day has been recommended. However, more intensive treatment protocols suggest administering 500 mg of thiamine hydrochloride by intravenous infusion, three times daily for 2–3 days. This is followed by an additional 250 mg of thiamine administered once daily for 3–5 days or until clinical symptoms improve [22].

Oral thiamine supplementation is not considered reliable and is generally not recommended in the acute management of WE. Due to potential malabsorption issues in high-risk patients, parenteral administration remains the preferred route [7].

It should be noted that thiamine should be given before or concurrently with glucose-containing solutions. This is crucial because glucose metabolism can further deplete thiamine levels, potentially exacerbating neurological symptoms and worsening the patient’s condition [23].

Most patients diagnosed with WE require hospital admission to ensure proper monitoring and intravenous thiamine administration. Continuous clinical assessment is essential to evaluate the patient’s response to treatment and determine the duration of therapy. Failure to administer adequate thiamine doses in a timely manner can lead to severe complications, including Korsakoff syndrome, a chronic and debilitating neuropsychiatric condition [7].

Despite thiamine treatment, WE carries significant morbidity and mortality. Most neuro-ophthalmic symptoms improve within weeks, with nystagmus often regressing within hours of supplementation in two-thirds of cases [24,25]. However, residual ataxia and neurological deficits may persist. The prognosis for visual acuity is generally favorable, but cases of permanent visual impairment have been reported [26].

Although the confusional state improves with intravenously administer thiamine, memory deficits often persist [15]. Some patients fail to recover fully and may develop Korsakoff psychosis, requiring long-term institutionalization. Less than 10% of patients with Korsakoff syndrome achieve full functional recovery [21]. Chronic neurological deficits, including ataxia, nystagmus, and cognitive impairment, can significantly impact the quality of life. Long-term follow-up studies are lacking, but anecdotal reports suggest premature mortality in affected individuals [27].

## 6. Optic Nerve Involvement in WE

Involvement of the optic nerve in WE is evidenced by changes in the optic disc and retina. Optic nerve disc edema and retinal hemorrhages were first described in Wernicke’s original report [28]. Edema of the optic disc is a rare phenomenon; it was found in 2 of 52 (4%) WE patients studied by De Wardener et al. [29]. Retinal hemorrhages are also uncommon and may be underreported due to limited physician experience with ophthalmoscopy [30].

Thiamine deficiency leads to the accumulation of pyruvic acid and lactic acid, which cause insufficient production of energetic adenosine-5′-triphosphate, leading to cytotoxic edema and cell death. Another mechanism is increased intracranial pressure. This leads to increased pressure around the optic nerves and consequent swelling around them and blocked axoplasmic flow. This ultimately leads to necrosis of nerve cells and myelin structures [31].

Bilateral optic disc swelling with peripapillary and diffuse retinal hemorrhages can be observed initially. With thiamine supplementation, these symptoms typically improve within one to two weeks, and complete resolution may occur after three weeks [32]. However, some cases of vision loss have been reported even in patients with normal-appearing optic discs [33].

Various ophthalmic abnormalities can occur in WE, affecting both the efferent and afferent visual systems. Nystagmus is the most common presenting symptom, typically manifesting as gaze-evoked horizontal nystagmus [15,20]. It is considered the earliest sign of thiamine deficiency [34]. When nystagmus is accompanied by an inability to maintain gaze, it suggests involvement of the nucleus prepositus hypoglossi [35].

Other ophthalmic manifestations may include bilateral paralysis of the adductor muscles, paired horizontal or vertical gaze palsies [36,37], and unilateral or bilateral internuclear ophthalmoplegia [38,39]. Additionally, bilateral vestibulo-ocular reflex dysfunction may be present [40].

Alterations in pupil size and reactivity to light [14,37], light-near dissociation [41], impaired convergence [42], contraction of the near reflex [43], and eyelid drooping (ptosis) [37] have also been reported.

## 7. Optical Coherence Tomography in Diagnosing Optic Neuropathy

OCT is a non-invasive imaging technology that utilizes low-coherence interferometry to generate high-resolution, three-dimensional images of biological tissues. Spectral-domain OCT (SD-OCT) provides an image resolution of 3 to 5 microns (µm) within a 50–600 µm range, offering a resolution approximately 1000 times greater than that achieved by MRI. Some SD-OCT platforms incorporate enhanced depth imaging (EDI), while the latest SS-OCT technology enables faster scan times and improved visualization of deeper retinal and choroidal layers [44,45].

In WE, OCT has emerged as a valuable tool for detecting optic neuropathy and retinal changes associated with thiamine deficiency. It allows for the quantitative assessment of key parameters, such as the peripapillary retinal nerve fiber layer (pRNFL) and the ganglion cell layer (GCL), which may be affected by neurodegeneration in WE. These measurements can be compared with normative databases to assess potential optic nerve involvement [46].

The pRNFL consists of axons originating from deeper-lying retinal ganglion cells, and its thickness serves as a crucial biomarker for optic nerve health. In WE, alterations in pRNFL thickness may reflect underlying neurodegenerative changes, with potential manifestations such as optic disc edema in the acute phase and optic nerve atrophy in later stages [44]. Additionally, subclinical changes in retinal structure observed via OCT may provide insights into the extent of neurological impairment in WE, further supporting its role in early diagnosis and disease monitoring.

## 8. Illustrative Case Example

A 23-year-old woman at 14 weeks of gestation presented with persistent vomiting since the 7th week of pregnancy. She had no history of malnutrition, restrictive diets, bariatric surgery, or gastrointestinal disorders. She was not taking any medications or supplements prior to admission. There was no prior history of hospitalizations or consultations related to her condition.

On admission, the patient exhibited severe neurological symptoms, including progressive visual impairment with visual acuity reduced to finger counting at close range, nystagmus, oculomotor disturbances, dizziness, memory deterioration, and increasing lower-limb paresis. Due to the patient’s poor overall condition and severely reduced visual acuity, visual field testing could not be performed at that time. Laboratory tests revealed significantly elevated levels of alanine aminotransferase (ALT, 1517 U/L; norm: 10–35 U/L), aspartate aminotransferase (AST, 534 U/L; norm: 10–35 U/L), gamma-glutamyltransferase (GGTP, 234 U/L; norm: 6–42 U/L), lipase (289 U/L; norm: 7–60 U/L), amylase 104 U/L (norm 28–100 U/L), and total bilirubin (3.05 mg/dL; norm: 0.10–1.20 mg/dL). No other significant abnormalities were found in laboratory tests.

The initial diagnostic approach considered hyperemesis gravidarum as the primary cause of her condition. However, given the presence of severe neurological deficits, other potential etiologies, including infectious, metabolic, and structural neurological disorders, were investigated. HBV, HCV, EBV, and CMV infections were ruled out through serological testing. Abdominal ultrasound showed no evidence of hepatobiliary pathology or acute abdominal conditions. Cerebrospinal fluid examination ruled out neuroinfections and acute inflammatory demyelinating polyneuropathy.

**Figure 1:**
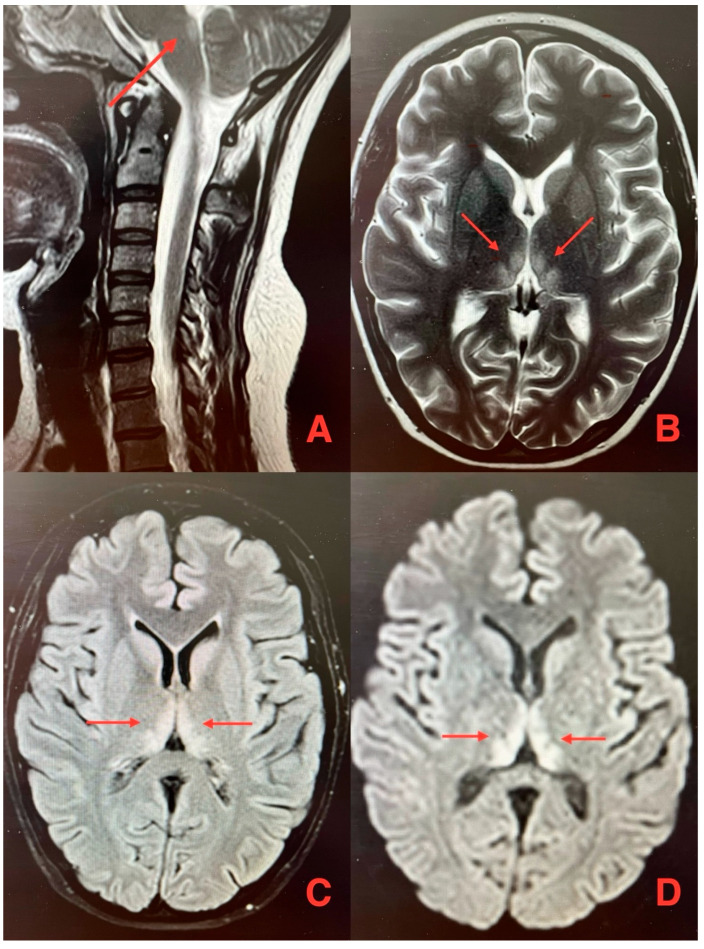
Magnetic resonance images of the brain show hyperintense lesions in the area of the third ventricle–arrows (T2-weighted sequence) (**A**) and in the medial area of both thalamuses (T2-weighted sequence (**B**), FLAIR sequence (**C**), and DWI sequence (**D**)) (author’s archive).

Brain MRI revealed symmetrical hyperintense lesions in the posterior medial thalamus and posterior periventricular region of the medulla oblongata on T2-weighted and FLAIR sequences, with restricted diffusion and contrast enhancement, findings which are characteristic of WE (Figure 1).

**Figure 2:**
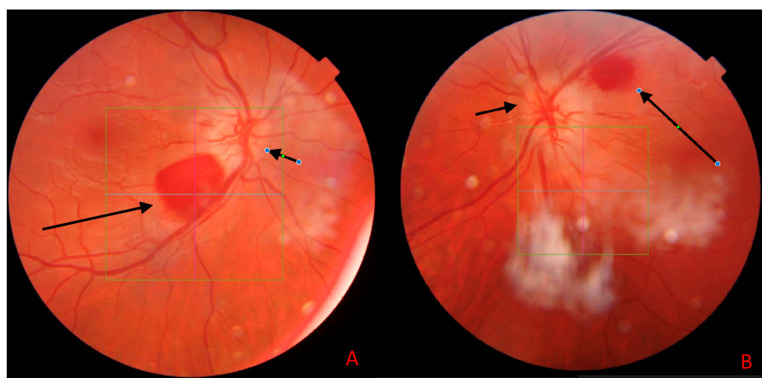
The color image of the fundus of the right eye (**A**) and the left eye (**B**) obtained with swept-source optical coherence tomography (SS-OCT) Triton during the first visit. The pale, swollen optic disc in the right and left eye (short arrows). Round preretinal hemorrhage in the central retina in the right eye (**A**) located above inferior retinal vessels (long, black arrow) and in the central retina in the left eye (**B**) located below the superior retinal vessels (long, black arrow). The white cloudy areas visible in the image represent artifacts and are not indicative of any retinal pathology (author’s archive).

SS-OCT was performed using a system with a central wavelength of 1050 nm (near-infrared), providing an axial resolution of 2.6 µm, a transverse resolution of 14 µm, a scan width of 12 mm, and a scanning speed of 370,000 A-scans per second. The use of near-infrared light reduces scattering and enables enhanced visualization of deep ocular structures, such as the posterior vitreous cortex and the choroid. Unlike spectral-domain OCT, the swept-source technology does not require a spectrometer, thereby avoiding limitations associated with nonlinear sensitivity profiles and allowing for improved image quality across both vitreous and choroidal layers. OCT demonstrated hyperreflectivity of the peripapillary area, increased peripapillary retinal nerve fiber layer (pRNFL) thickness during the acute phase, and subsequent thinning of this layer on follow-up imaging (Figure 2).

Thiamine levels were measured before treatment initiation and found to be significantly reduced (6.6 µg/L; reference range: 28 do 85 µg/L), confirming the diagnosis of WE. Given the absence of other metabolic disorders and the characteristic imaging findings, alternative neurological conditions, such as multiple sclerosis, neurodegenerative diseases, and ischemic stroke, were considered unlikely.

The diagnosis of WE was established clinically based on the Caine criteria, with the patient fulfilling all four of the following: dietary deficiency due to prolonged hyperemesis gravidarum, oculomotor disturbances, cerebellar dysfunction, and altered mental status. The clinical diagnosis was further supported by laboratory findings of significantly reduced serum thiamine levels and by characteristic MRI features, including symmetric hyperintensities in the medial thalami and periaqueductal gray matter on T2-weighted and FLAIR sequences.

The patient was treated with parenteral thiamine at a dose of 100 mg daily. Supportive therapy, including intravenous hydration, was also administered. Improvement in neurological symptoms was observed within 2 weeks, with progressive recovery of visual function and motor coordination over the following weeks. 

**Figure 3:**
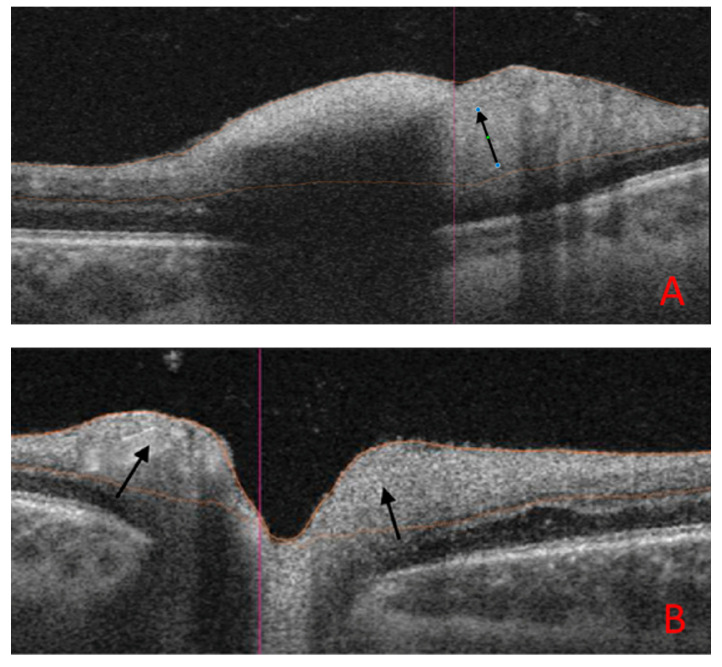
The B-scan image of the optic nerve disc in the right eye (**A**) and in the left eye (**B**), obtained with optical coherence tomography (OCT) TRITON during the first days of hospitalization. Hyper-reflectiveness and swelling of the optic disc correspond to the swollen nerve fiber layer of the optic nerve disc and peripapillary area (black arrows). The orange line marks the thickened nerve fibre layer of the pRNFL, the red line indicates the vertical scan of the optic nerve disc. (author’s archive).

**Figure 4:**
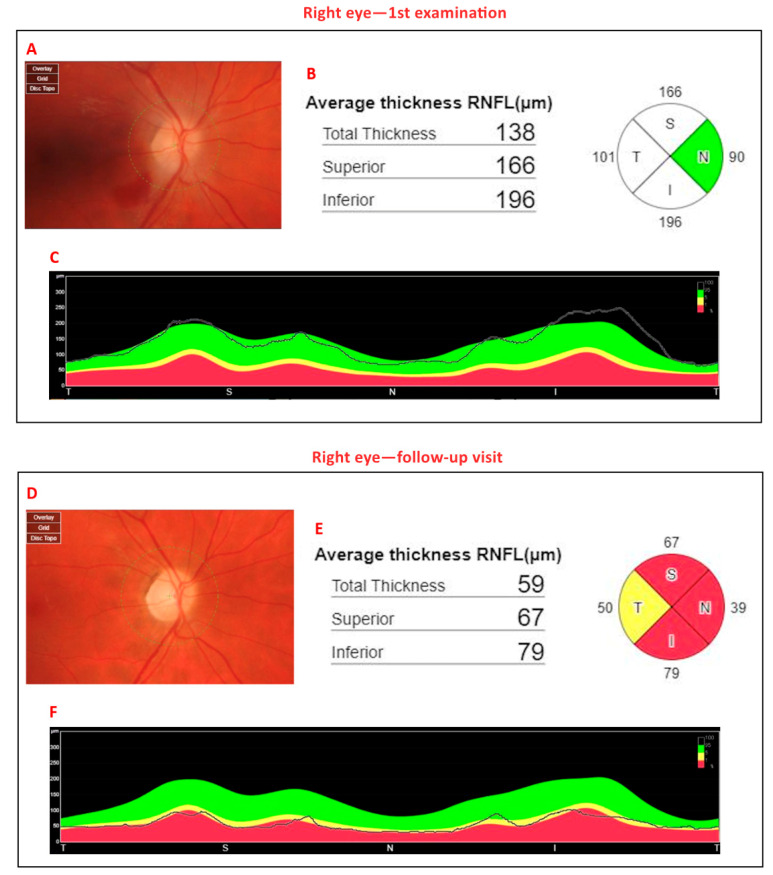
Diagram of peripapillary retinal nerve fiber layer (pRNFL) of right eye. (**A**) The color image of the right optic disc. The pale, swollen optic disc and preretinal hemorrhage located next to the nasal margin of the optic disc are visible. (**B**): Circle diagram of pRNFL (thickness of pRNFL; total thickness = 138 µm, superior thickness = 166 µm, inferior thickness = 196 µm). (**C**): The black line determines thickening of the pRNFL layer in four sectors of the optic disc (T—temporal, S—superior, N—nasal, I—inferior). (**D**): The color image of the right optic disc. The pale, atrophic optic disc is visible. (**E**): Circle diagram of pRNFL (thickness of pRNFL; total thickness = 59 µm, superior thickness = 67 µm, inferior thickness = 79 µm). (**F**): The black line determines thinning of the pRNFL layer in four sectors of the optic disc. Thickness of pRNFL was obtained with swept-source optical coherence tomography (SS-OCT) Triton during the first examination (**A**–**C**) and during the follow-up visit 5 months later (**D**–**F**). The segmentation and thickness measurements were generated entirely through the device’s automated analysis software, without manual modification (author’s archive).

**Figure 5:**
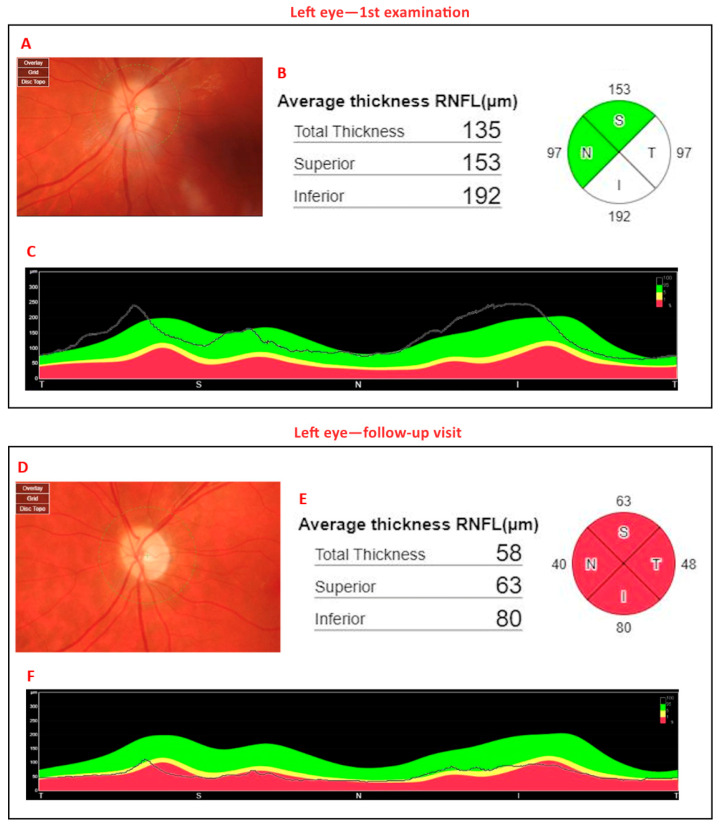
Diagram of peripapillary retinal nerve fiber layer (pRNFL) of left eye. (**A**) The color image of the left optic disc. The pale, swollen optic disc is visible. (**B**) Circle diagram of pRNFL (thickness of pRNFL; total thickness = 135 µm, superior thickness = 153 µm, inferior thickness = 192 µm). (**C**) The black line determines thickening of the pRNFL layer in four sectors of the optic disc (T—temporal, S—superior, N—nasal, I—inferior). (**D**) The color image of the left optic disc. The pale, atrophic optic disc is visible. (**E**) Circle diagram of pRNFL (thickness of pRNFL; total thickness = 58 µm, superior thickness = 63 µm, inferior thickness = 80 µm). (**F**) The black line determines thinning of the pRNFL layer in four sectors of the optic disc. Thickness of pRNFL was obtained with swept-source optical coherence tomography (SS-OCT) Triton during the first examination (**A**–**C**) and during the follow-up visit 5 months later (**D**–**F**). The segmentation and thickness measurements were generated entirely through the device’s automated analysis software, without manual modification (author’s archive).

Figure 3 shows B-scans of SS-OCT in a patient with WE through hyperreflectivity of the peripapillary area, and Figure 4 and Figure 5 show a quantitative assessment of pRNFL in both eyes in this patient in all sectors during the acute phase of WE; pRNFL is elevated in both eyes in the acute phase of WE (Figure 3A,B, Figure 4A–C and Figure 5A–C).

**Figure 6:**
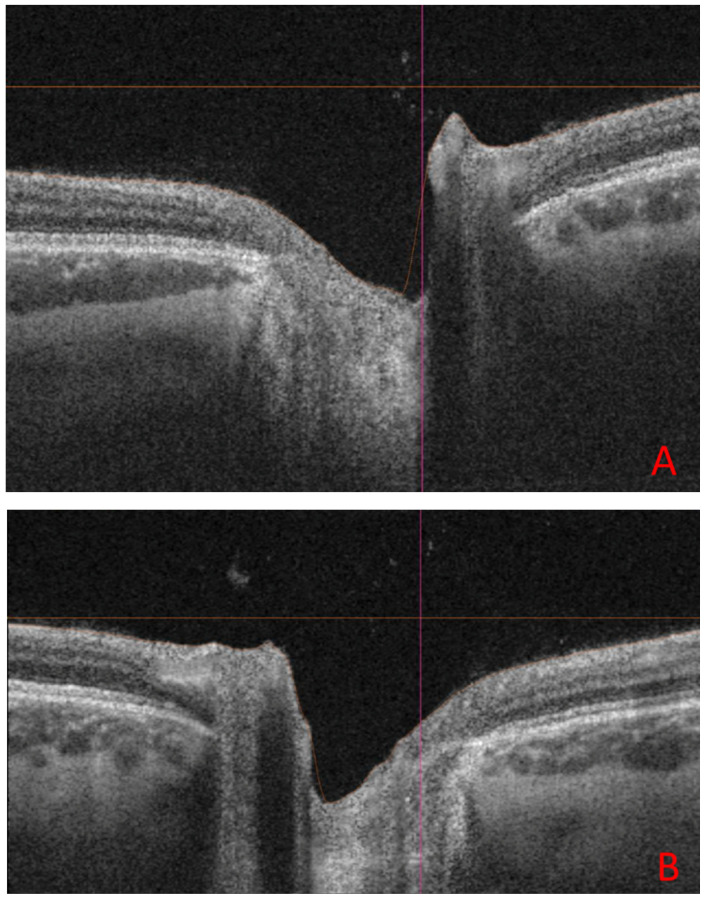
The B-scan image of the optic nerve disc in the right eye (**A**) and in the left eye (**B**) obtained with optical coherence tomography (OCT) TRITON during the last visit (5 months after the first visit). Physiological depression in the right eye. The orange line marks the boundary of thickened nerve fibre layer of the pRNFL, the red line indicates the vertical scan of the optic nerve disc. The horizontal orange line indicates the horizontal scan of the optic nerve disc. (author’s archive).

At a 5-month follow-up visit, the patient had fully regained neurological function, with normal color vision and no residual motor deficits. The OCT scan also allowed the monitoring of pRNFL thickness over time. Follow-up OCT scans showed the resolution of optic disc swelling; however, the optic nerve appeared paler, (Figure 6A,B), although the quantitative assessment of the pRNFL showed a significant reduction in this layer in all sectors in both eyes (Figure 4D–F and Figure 5D–F).

**Figure 7:**
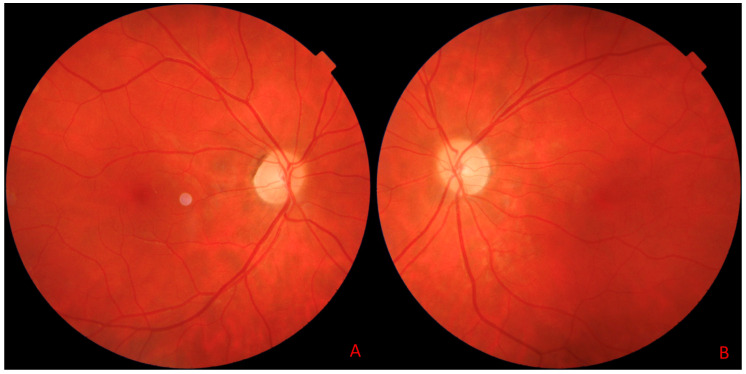
The color image of the fundus of the right eye (**A**) and of the left eye (**B**) obtained with swept-source optical coherence tomography (SS-OCT) Triton during the last visit. Paler, round optic disc in the right eye, without swelling (author’s archive).

Despite good visual acuity and unchanged color vision in both eyes at the 5-month follow-up visit, OCT enabled ongoing monitoring of the optic disc and provided prognostic insight into its early atrophic changes, as evidenced by the paler, round optic disc appearance in the right eye (Figure 7).

**Figure 8:**
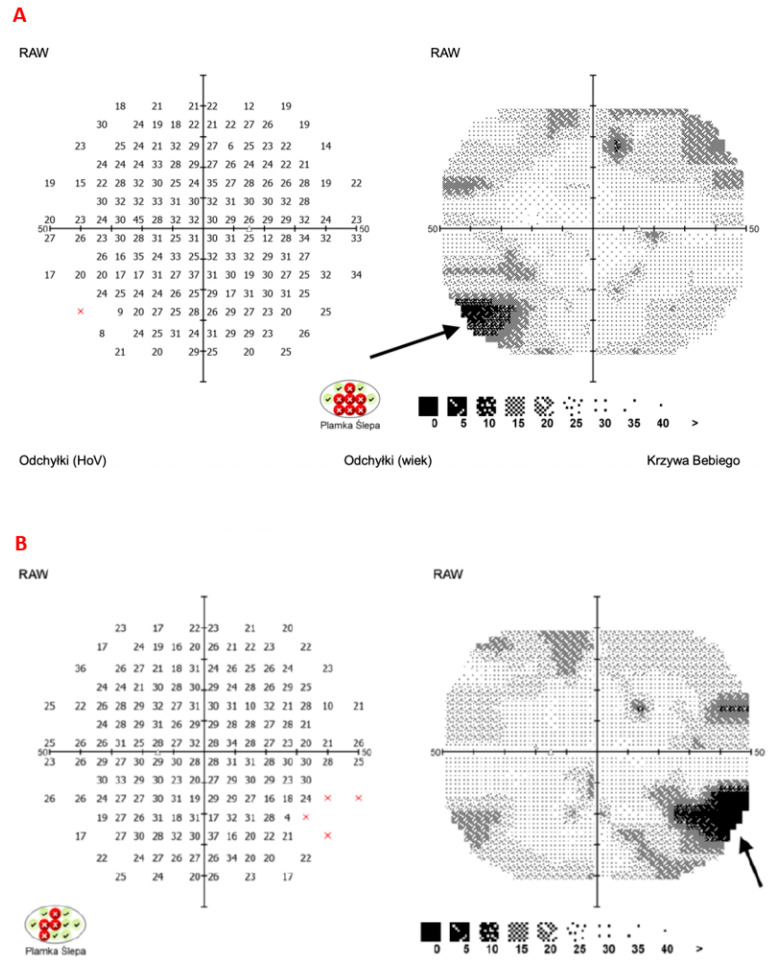
Visual fields of the right eye (**A**) and left eye (**B**) obtained using automated perimetry (F 50-2, Optopol Technology, PTS 925) during the last follow-up visit. In A and B black arrows indicate peripheral visual field defects. Red “×” in the figure is the size of blind spot in right and left eye (author’s archive).

Visual field testing performed at the follow-up visit revealed no central scotomas; only subtle peripheral defects were observed, as indicated by the arrows in the visual field image (Figure 8).

**Figure 9:**
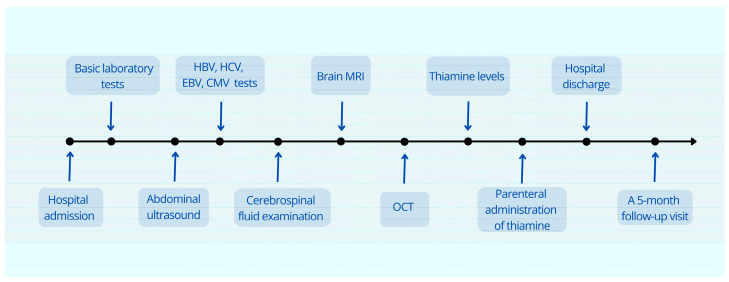
Schematic flow diagram illustrating the diagnostic and follow-up process for Wernicke’s encephalopathy (WE) secondary to thiamine deficiency.

She was advised to continue oral thiamine supplementation throughout pregnancy and postpartum to prevent recurrence. Nutritional counseling was provided to ensure adequate dietary intake of thiamine-rich foods. Long-term neurological and ophthalmological follow-up was recommended to monitor for potential late complications (Figure 9).

## 9. Discussion

WE, which was first described by Carl Wernicke in the 19th century, is a neuropsychiatric disorder resulting from thiamine deficiency [23]. The presented case highlights the risk of WE in pregnancy, particularly in the setting of prolonged vomiting, which significantly impairs thiamine absorption. Pregnancy itself is a hypermetabolic state with increased thiamine requirements, further predisposing individuals with hyperemesis gravidarum to deficiency [9]. It has been established that persistent vomiting for approximately three weeks can deplete the body’s thiamine stores, leading to the onset of neurological symptoms [47]. In our patient, neurological deficits, including progressive visual impairment, nystagmus, oculomotor dysfunction, and lower-limb paresis, developed after several weeks of persistent vomiting, highlighting the importance of early recognition and intervention.

The diagnosis of WE is challenging, as the classic triad of ophthalmoplegia, ataxia, and confusion is observed in only a minority of cases at presentation. A postmortem study of 131 patients with WE found that only 16% exhibited all three features [48]. In our patient, severe visual disturbances, oculomotor dysfunction, progressive neurological impairment, low thiamine levels, and characteristic MRI findings enabled an early and accurate diagnosis.

Early recognition and prompt thiamine supplementation are crucial, as WE symptoms are reversible in the early stages. The diagnosis is supported by characteristic MRI findings, and rapid clinical improvement with intravenous thiamine confirms it. However, maternal and fetal outcomes remain concerning, with studies showing that only 50% of pregnancies complicated by WE result in the birth of a healthy infant [47]. In untreated cases, the risk of severe maternal complications, including permanent neurological deficits, progression to Korsakoff syndrome, and mortality rates of 10–20%, highlights the critical need for timely intervention. Additionally, WE has been associated with an increased risk of miscarriage, preterm birth, and intrauterine growth restriction [49]. Fortunately, in our patient, timely diagnosis and parenteral thiamine treatment resulted in a favorable maternal and fetal outcome. The pregnancy progressed without complications, and follow-up confirmed normal fetal development. At delivery, both mother and newborn were in good health, highlighting the importance of early intervention in preventing adverse outcomes associated with WE.

Ophthalmological involvement in WE has been well documented, with ocular motility disturbances and fundoscopic abnormalities frequently reported [50,51]. The pathogenesis of optic nerve involvement in thiamine deficiency is related to mitochondrial dysfunction, a mechanism common to both toxic and genetic optic neuropathies. These conditions typically present with bilateral, painless central vision loss and dyschromatopsia, reflecting the high metabolic demand of retinal ganglion cell axons in the optic nerve head. The ganglion cell layer is especially vulnerable due to its reliance on mitochondrial oxidative phosphorylation for axonal transport [52].

Mitochondrial dysfunction due to thiamine deficiency is believed to initially cause swelling and microhemorrhages within the retinal nerve fiber layer, with subsequent optic disc edema developing if the damage persists [53]. Nutritional optic neuropathies are classified within acquired mitochondrial optic neuropathies due to shared pathophysiological mechanisms. Clinically, these syndromes often resemble toxic optic neuropathies, complicating diagnosis. Previous OCT studies in chronic alcohol and tobacco users, who are commonly affected by thiamine deficiency, have shown significant pRNFL thinning, with only the nasal quadrant spared [54].

In our case, OCT imaging showed hyperreflectivity of the peripapillary area and increased pRNFL thickness during the acute phase of WE, indicating early mitochondrial dysfunction and axonal swelling. These findings align with previous studies, where early-stage nutritional and toxic–metabolic optic neuropathies show mitochondrial accumulation in RGC axons, leading to optic disc edema. Over time, chronic damage results in optic disc pallor, especially in the temporal sector, with progressive pRNFL thinning.

Notably, the patient had no known pre-existing ocular or neurological conditions that could influence retinal nerve fiber layer thickness or optic nerve morphology, ensuring that the observed OCT changes were attributable to the acute episode of WE.

In our case, OCT imaging revealed dynamic changes in the RNFL, reflecting the pathological evolution of optic neuropathy in the course of WE. At the initial examination, the RNFL was diffusely thickened, which we interpret as edema resulting from acute hypoxia and nutritional deficiency affecting the optic nerve head and peripapillary retina. At the 5-month follow-up, OCT revealed clear RNFL thinning, consistent with axonal loss and atrophy of the optic nerve. These findings support the hypothesis that structural changes in the RNFL evolve from early swelling to late-stage atrophy, mirroring the clinical course of the disease. Similar OCT patterns have been reported in other nutritional optic neuropathies, where thinning of the RNFL begins in the papillomacular bundle and progressively involves all quadrants [55,56]. This further supports the utility of swept-source OCT in detecting and monitoring optic nerve involvement in WE, even before fundoscopic changes become apparent.

At the five-month follow-up, our patient demonstrated complete functional visual recovery, yet OCT still detected a reduction in pRNFL thickness, indicating ongoing structural changes despite the resolution of clinical symptoms. This underscores the importance of OCT as a sensitive tool for detecting subclinical optic nerve atrophy and monitoring disease progression. However, the generalizability of these findings is limited, especially given the rare and specific etiological context of WE secondary to hyperemesis gravidarum. Additional potential confounding factors, such as dehydration and electrolyte imbalances commonly present in HG, may independently influence OCT measurements and should be taken into account. Further studies involving larger, more diverse patient populations are warranted to validate these preliminary observations and better define the diagnostic utility of OCT in WE across various clinical contexts.

## 10. Conclusions

Patients with suspected WE should be consulted ophthalmologically for optic nerve neuropathy. OCT is a useful, non-invasive examination that can help make a diagnosis of optic nerve neuropathy. OCT enables the diagnosis and monitoring of patients with WE optic nerve neuropathy in terms of the assessment of RNFL thickness and prognosis of visual acuity. Serum thiamine levels should be tested in these patients and, if deficiency is found, supplementation should be implemented as soon as possible to prevent the development of optic nerve neuropathy and WE.

## Data Availability

The data supporting the reported results can be provided upon request to the corresponding author.

## Abbreviations

The following abbreviations are used in this manuscript:

ALT	Alanine Aminotransferase
AST	Aspartate Aminotransferase
CMV	Cytomegalovirus
EBV	Epstein–Barr Virus
FLAIR	Fluid-Attenuated Inversion Recovery (MRI sequence)
GGTP	Gamma-Glutamyl Transpeptidase
HBV	Hepatitis B Virus
HCV	Hepatitis C Virus
MRI	Magnetic Resonance Imaging
OCT	Optical Coherence Tomography
pRNFL	Peripapillary Retinal Nerve Fiber Layer
SD-OCT	Spectral-Domain Optical Coherence Tomography
SS-OCT	Swept-Source Optical Coherence Tomography
WE	Wernicke’s Encephalopathy
